# Predicting Nucleosome Positioning Based on Geometrically Transformed Tsallis Entropy

**DOI:** 10.1371/journal.pone.0109395

**Published:** 2014-11-07

**Authors:** Jing Wu, Yusen Zhang, Zengchao Mu

**Affiliations:** School of Mathematics and Statistics, Shandong University at Weihai, Weihai, China; The University of Hong Kong, Hong Kong

## Abstract

As the fundamental unit of eukaryotic chromatin structure, nucleosome plays critical roles in gene expression and regulation by controlling physical access to transcription factors. In this paper, based on the geometrically transformed Tsallis entropy and two index-vectors, a valid nucleosome positioning information model is developed to describe the distribution of A/T-riched and G/C-riched dimeric and trimeric motifs along the DNA duplex. When applied to train the support vector machine, the model achieves high AUCs across five organisms, which have significantly outperformed the previous studies. Besides, we adopt the concept of relative distance to describe the probability of arbitrary DNA sequence covered by nucleosome. Thus, the average nucleosome occupancy profile over the S.cerevisiae genome is calculated. With our peak detection model, the isolated nucleosomes along genome sequence are located. When compared with some published results, it shows that our model is effective for nucleosome positioning. The index-vector component 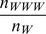 is identified to be an important influencing factor of nucleosome organizations.

## Introduction

As the basic structural unit of eukaryotic chromatin, nucleosome is composed of DNA with 147 bp wrapped 1.65 turns around a protein complex of eight histones. A stretch of around 10–100 bp free DNA termed linker DNA joined two neighboring nucleosomes together (Luger et al. 1997; Richmond and Davey 2003). The presence (absence) of nucleosomes directly (indirectly) affects a variety of processes of life, including recombination, replication, centromere formation and DNA repair.

The developments of the high-thoughput techniques such as chromatin immunoprecipitation (CHIP) coupled with microarrays (CHIP-chip) and CHIP coupled with sequencing techniques (CHIP-Seq) have enabled landmark genome-wide studies of nucleosome positions for several model organisms, like Yeast, Caenorhabditis elegans, Drosophila and Human, which allow the researchers to establish models for nucleosome positioning as well as explore the internal relations between them and the expression and regulation among the whole genome.

Nucleosome formation along genome depends on multiple factors, including perference of DNA sequence, physical constraints and epigenetic factors like activities of ATP-dependent remodeling complex. Thus, the precise mechanism of nucleosome formation remains unknown. In the initial research of nucleosome, some researchers have demonstrated that AA/TT/TA have a periodility of 10.4 bp along the genome, poly-A contents and some conserved sequence motifs are important signals for nucleosome positioning. A few computational models were also proposed based on the preference of DNA sequences itself. Segal et al. established a probabilistic model to characterize the possibility that one DNA sequence is occupied by nucleosome [5]. Peckham et al. introduced a supervised classification algorithm: support vector machine to do the binary classification [8]. Yuan and Liu proposed an N-score model to discriminate nucleosome and linker DNA sequences with wavelet transformation and logarithmic regression in 2008 [6]. In the same year, a web-interface called ‘nuScore’ was developed for estimating the affinity of histone core to DNA and predicition of nucleosome positioning. However, the success achieved by these models are limited, some research institutions have begun to study the structural characteristics of DNA sequences as well as the conformation mechanism of nucleosomes. Some physicochemical properties of nucleosome have shown their significant influence on the nucleosome positioning, such as tilt, twist and free energy, Tolstorukov et al. [24], Miele et al. [20], Morozov et al. [26] have done excellent work focusing on the role that structural features play in the nucleosome positioning. Therefore, it is very necessary to systematically analyze the different structural characteristics as well as identifying the structural characteristics that play roles in the formation of nucleosome. Furthermore, it is desirable to integrate those structural features that contribute to the formation of nucleosome to improve the prediction of nucleosome.

In this paper, we proposed three main models: nucleosome positioning information model, nucleosome occupancy model, peak detection model to form the complete nucleosome positioning model. The nucleosome positioning information model was developed based on the geometrically transformed Tsallis entropy combined with two index-vectors. We showed that our model has better performance in the discrimination of known nucleosomal and linker DNA sequences across five organisms (Human, Medaka, Nematode, Candida and Yeast) compared with the previous work of Segal et al. [5], [9], [22], Miele et al. [20], Gupta et al. [21] and Zhang et al. [16]–[19]. Moreover, we adopted the concept of relative distance to donate the potential that one sequence belongs to nucleosomal DNA. The average nucleosome occupancy profile over the S.cerevisiae genome was calculated and compared with the previous work of Kaplan et al. [9], Segal et al. [5], good correlations (correlation coefficients of 0.6858 and 0.7626, respectively) were shown. Furthermore, by identifying the real peaks with peak detection model, we located the isolated nucleosomes along the yeast genome. By comparing with some published maps [5], [6], we demonstrate that our model is simple and efficient for predicting nucleosome positions along genome.

## Materials and Methods

### Genomic DNA and nucleosome positioning data

The genome sequences were downloaded from the S.cerevisiae Genome Database (http://www.yeastgenome.org/), which correspond to the sequences of Chromosomes *I*-*XVI* on January 2006. The data of five organisms (Human, Medaka, Nematode, Candida and Yeast) used to validate the performance of nucleosome positioning information model was obtained from the published work of Tanaka et al. [1]. In their work, 100 nucleosomal and 100 linker DNA sequences whose length are between 100 bp and 200 bp were extracted from the genome-scale nucleosome map. These processes were repeated 10 times. Besides, the data referring to nucleosome positioning was collected from the published works (Kaplan et al. [9], Segal et al. [5], Yuan et al. [4], [6], [10], Lee et al. [7], Mavrich et al. [11], Albert et al. [12]).

### Tsallis entropy theory

Tsallis entropy theory was first introduced in 1988 by Constantino Tsallis [2]. It can be described as follows:

In a system, set 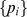 be a discrete set of probabilities with the condition: 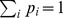. 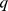 is a real parameter sometimes called entropic-index. The Tsallis entropy of the system is defined by
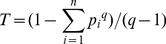
(1)


In the information theory, entropy is a measure of the uncertainty and an index that shows the state of one material system. Since the year 2000, an increasingly wide spectrum of natural, artificial and social complex systems have been identified which confirm the predictions and consequences that are derived from this nonadditive entropy. Here, the Tsallis entropy theory was used to measure the conservativeness of information of nucleosomal and linker DNA.

To describe the nucleosome positioning information in arbitrary DNA sequence, the geometrically transformed Tsallis entropy component 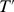 was introduced.
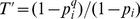
(2)where 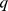 is a real number and 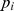 is a probability between 0 and 1.

### Nucleosome positioning information model

Inspired by the pioneering work of Trifonov [15] that AT-riched and GC-riched dimeric and trimeric motifs were contributed to nucleosome organization, we further explored the role that A/T-riched and G/C-riched dimeric and trimeric motifs play in nucleosome organization.

Given four nucleotides (A, T, G, C), we calculated the Pearson correlation coefficients between the nucleosome occupancy and the single-nucleotide frequencies across five organisms, respectively (Table 1). We noticed that expect for Medaka, the other four organisms shared the same role: A and T are both negatively related with nucleosome occupancy while C and G are both positively related. The result of Yeast is consistent with the findings that the single-nucleotide frequencies C+G were nucleosome forming features while A+T were nucleosome inhibiting features in Peckham's work [8]. This may be the reason for the phenomenon that the AT-rich intergenic regions in S.cerevisiae are nucleosome-free [25]. Tanaka and Nakai [1] have pointed out that the nucleosomal DNAs in Medaka were quite different from other four species. In summary, A and T shared the same relation with nucleosome occupancy across five organisms, so were C and G. Then, the four nucleotides can be divided into two classes: W and S (where W is A or T, S is C or G). In this way, each DNA sequence was converted to a vector composed by S and W.

**Table 1:** The correlation coefficients between four nucleotides and the nucleosome occupancy across five organisms.

Organisms	A	T	G	C
Human	−0.2419	−0.1861	0.2483	0.2356
Medaka	0.0511	0.0119	−0.0229	−0.0453
Nematode	−0.1749	−0.2394	0.2526	0.2635
Candida	−0.1382	−0.1093	0.1547	0.2098
Yeast	−0.2276	−0.1997	0.3299	0.2962

We considered a DNA sequence read and its reverse complement together in a 5′ to 3′ fashion, the occurrences of k-mers, 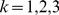 were counted, donated by 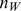, 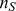, 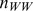, 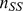 and 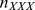 (where X is W or S). Thus, we proposed two index-vectors:
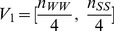
(3)

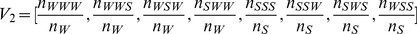
(4)



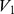
 extracts the frequencies of A/T-riched and G/C-riched dimeric motifs along each sequence. 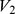 depicts the relative frequency of A/T-riched (G/C-riched) trimeric motifs when A/T (C/G) appears. Furthermore, combined with the geometrically transformed Tsallis entropy, two 4-dimensional vector 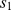 and 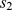 were constructed to represent the conservation of A/T-riched and G/C-riched motifs along each DNA duplex, respectively.
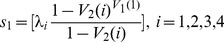
(5)

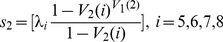
(6)


Here, 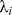 is a coefficient donated to illustrate the relative conservation between the distributions of trinucleotides along one DNA strand and its reverse complement. Suppose that the distribution of one trinucleotide along a single DNA strand is 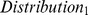, while the distribution along its reverse complement is 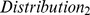. Next, we consider the relationship between 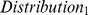 and 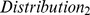 for all eight trinucleotides.

When the trinucleotide and its reverse complimentary element are the same, such as 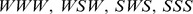, which means that the locations of these trinucleotides along one DNA strand can determine the positions that these trinucleotides along the other DNA strand totally. We consider the two distributions of these trinucleotides along DNA duplex are completely conservative and 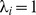. For the other four trinucleotides, 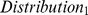 and 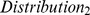 along DNA duplex are independent, so 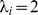.

However, the lengths of nucleosomes extracted by different ways are different for different organism, even the same organism. In order to eliminate the impact of length difference, we took the length of nucleosome sequences in Saccharomyces cerevisiae (147 bp) as a standard.

Therefore, the nucleosome positioning information model can be established as:
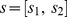
(7)

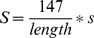
(8)


Here, 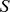 can be used to describe the conservation of A/T-riched and G/C-riched dimeric and trimeric motifs along arbitrary DNA duplex.

### Nucleosome occupancy model

We proposed a concept of relative distance to weight the potential that arbitrary DNA sequence belongs to nucleosomal DNAs. In this study, we constructed a training set consisting of the 1000 highest (nucleosome forming) and 1000 lowest (nucleosome inhibiting) scoring 50-bp fragments from chromosome 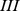 of the data set [4]. According to nucleosome positioning information model, the sequences in positive training dataset and negative training dataset can be translated into 8-component vectors, donated by 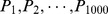 and 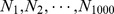, respectively.

In the Cartesian coordinate axis systems, Nandy [27] denoted 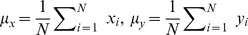 as the geometrical center (a weighted mean of the coordinate values of the representative points) of the points in a 2-D graph, where N represents the total number of points, 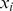 and 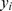 are the coordinates of the i-th point in the Cartesian coordinate system. By considering 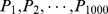 and 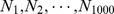 as the points in two high-dimensional Cartesian coordinate axis systems, we took their geometrical centers 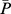 and 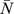 as the representative vector of the two systems, respectively.

Furthermore, the relative distance parameter for any given DNA fragment can be defined as:
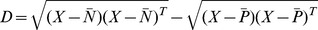
(9)where X is the 8-dimension vector corresponding to given DNA fragment.

Next, we sought to learn the average nucleosome occupancy along genomic sequence. Consider a genomic sequence S with n bases from the 5′− to 3′− end. A 147-bp sliding window was used to scan it from start to end in 1-bp step. Suppose the relative distance between the sequence in the 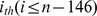 window to the negative training data and positive training data is 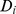. It is obvious that 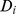 can measure the potential of starting a nucleosome at position i.

After applying the Range normalization transformation to 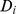, we calculated the average nucleosome occupancy of a basepair i of S covered by any nucleosome by defining the probability 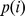, as follows:
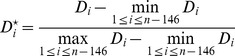
(10)

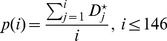
(11)

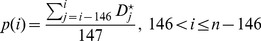
(12)

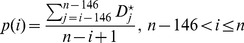
(13)


### Peak detection model

After mapping the average nucleosome occupancy profile, we identified the peaks as well-positioned properties indicating the positions of nucleosome. Suppose the nucleosome occupancy profile is M. After finding out all the ‘peaks’ along M with a sliding window of 147-bp in 10-bp step, we sought to identify the ‘real peaks’ with peak detection model.

Firstly, despite some latest researches have shown that there exist ‘fuzzy’ nucleosome, which means that two nucleosomes are ‘overlapping’ on the same location along DNA strands [23], our goal is to locate isolated nucleosomes. Based on this, if the distance between any two identified peaks is less than 147 bp, the peak with smaller value will be filtered out.

Secondly, according to the definition of 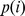, its value can give partial decision for the classification. We tried to train a threshold 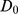. If the value of peak is less than 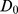, the peak will be identified as a ‘dummy peak’ and filtered out. Hence, the choose of 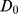 is crucial, as the smaller value is not sufficient for filtering out all ‘dummy peaks’, while the bigger value has too strong filtration effect. Here, we chose 0.5 as the optimal cutoff, which represents the random level.

After identifying all the ‘real peaks’, we denoted the position with the maximal value is 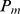, which is considered to be chromosomal coordinate. Then, the beginning 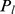 and ending 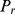 of this identified nucleosome are correspondingly determined as: 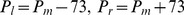, since the length of a well-positioned nucleosome in S.cerevisiae is 147 bp. By mapping the identified nucleosome positions onto the genomic sequence, the nucleosome organization graph can be obtained.

## Results

### Geometrically transformed Tsallis entropy analysis of the nucleosomal and linker DNAs

In this work, the geometrically transformed Tsallis entropy was raised to describe the nucleosome positioning information in nucleosomal and linker DNA fragments. The average values of 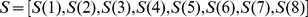 across five organisms (Human, Medaka, Nematode, Candida, Yeast) were listed in Table 2.

**Table 2:** A list of average geometrically transformed Tsallis entropy components S(i) in the nucleosomal and linker DNA regions of five organisms.

Organisms	Sequence type	S(1)	S(2)	S(3)	S(4)	S(5)	S(6)	S(7)	S(8)
Human	Nucleosomal DNAs	1.5194	2.5090	1.3346	2.5090	1.1310	2.5332	1.3785	2.5332
	Linker DNAs	2.2691	3.1800	1.6567	3.1800	1.4037	3.1585	1.6705	3.1585
Medaka	Nucleosomal DNAs	1.3469	2.5642	1.4102	2.5642	1.1535	2.5889	1.4514	2.5889
	Linker DNAs	1.7821	3.2486	1.7777	3.2486	1.5320	3.3119	1.8363	3.3119
Nematode	Nucleosomal DNAs	1.6418	2.4972	1.2648	2.4972	1.1182	2.5197	1.2839	2.5197
	Linker DNAs	2.3386	2.9107	1.4669	2.9107	1.3270	2.9254	1.4735	2.9254
Candida	Nucleosomal DNAs	1.6523	2.5048	1.2480	2.5048	1.0536	2.4575	1.2182	2.4575
	Linker DNAs	2.1823	2.8346	1.4203	2.8346	1.2208	2.7888	1.3927	2.7888
Yeast	Nucleosomal DNAs	1.5265	2.6196	1.2978	2.6196	1.1400	2.5952	1.2790	2.5952
	Linker DNAs	2.0145	2.7987	1.3849	2.7987	1.2600	2.7918	1.3698	2.7918

The average value of 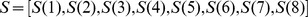 deciphers the average level of conservation of the distribution of A/T-riched and G/C-riched dimeric and trimeric motifs along DNA duplex. We noticed that the average value of S in nucleosomal DNA regions are all lower than that in linker DNA regions across five organisms. In other words, the distribution of A/T-riched and G/C-riched dimeric and trimeric motifs along nucleosomal DNA duplex was more conservative than that along linker DNA duplex. This result may be interpreted by the specific underlying interaction between the core histone octamer and DNA sequences in the structure of nucleosomes. As expected, the average value of vector 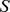 can apparently distinguish the nucleosomal and linker DNA sequences. The observation revealed that the geometrically transformed Tsallis entropy can efficiently extract the nucleosomal positioning information across five organisms.

### Evaluation of nucleosome positioning information model

As a supervised classification algorithm, SVM separates two or more groups according to the given characteristics [29]. The working theory is to map the data in training set onto a higher dimensional feature space. Then, the optimal plane separating the positive and negative examples can be obtained by finding the maximum margin from any point in the training set. The data in test set can be determined on which side of the separating plane by mapping it to the higher dimensional feature space.

Our study used the LIBSVM (http://www.csie.ntu.edu.tw/cjlin) for SVM classification [3]. In our application, the sequences were presented by the vector in function (8) and the two groups are ‘Nucleosomal DNAs’ and ‘Linker DNAs’. For the two parameters of LIBSVM, we set 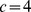 and 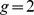 in this study.

We evaluated the quality of resulting classifier using a 5-fold crossvalidation procedure. In this procedure, the sequences both in positive and negative set will be divided into five subsets at random. A SVM is trained on 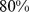 of the data (i.e. using 1600 sequences) and tested on the rest. Afterwards, the 1st, 2ed, 3rd, 4th and 5th set will be used as a test set in turn, while the rest four sets were retained as training set, which were used to train and construct a binary classification model. After obtaining the trained model, the sequences in the test set will be predicted with labels of 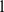 or 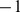, which means it being divided into the positive or negative set.

The performance of our model was measured by four parameters: total accuracy (Accuracy), the sensitivity (Sensitivity), positive predictive value (Precision) and Matthews correlation coefficient (MCC), defined as follows:
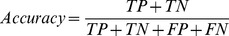
(14)

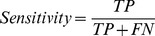
(15)

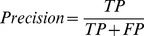
(16)

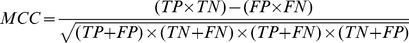
(17)


Where TP, TN, FP and FN represent the number of correctly predicted positive sequences, the number of correctly predicted negative sequences, the number of incorrectly predicted positive sequences and the number of incorrectly predicted negative sequences, respectively.

Another parameter used to evaluate the performance of our new model is the ROC curve (Relative Operating Characteristic curve), which plots the rate of true positives as a function of the rate of false positives for various classification thresholds. It is a comprehensive index to reflect the sensitivity and specificity of continuous variables. ROC curve sets the true positive rate as y-axis and the false positive rate as x-axis. The quality of a classifier can be evaluated by calculating the percentage(AUC) of the area under the ROC curve. If the value of AUC is 0.5, the experimental effect is equivalent to random separation, which means our work is meaningless, if between 0.5 and 0.7, this experiment is with poor effect. The value between 0.7 and 0.9 indicates good separation effect and above 0.9 is corresponding to excellent separation.

### Results of classifier based on nucleosome positioning information model compared with other publications

In Yoshiaki Tanaka's work [1], they compared the representative algorithms from three typical classes of prediction methods over the same dataset: Segal et al. [5], [9], [22] constructed their model mainly based on the 10-bp sequence periodicity. Miele et al. [20] studied the roles that physical properties played in determining nucleosome occupancy from yeast to fly. Gupta et al. [21] used the statistic of oligomer frequency to train SVM. In a recent study, Zhang et al. [17] trained SVM based on the dinucleotide absolute frequency of DNA sequence.

**Figure 1:**
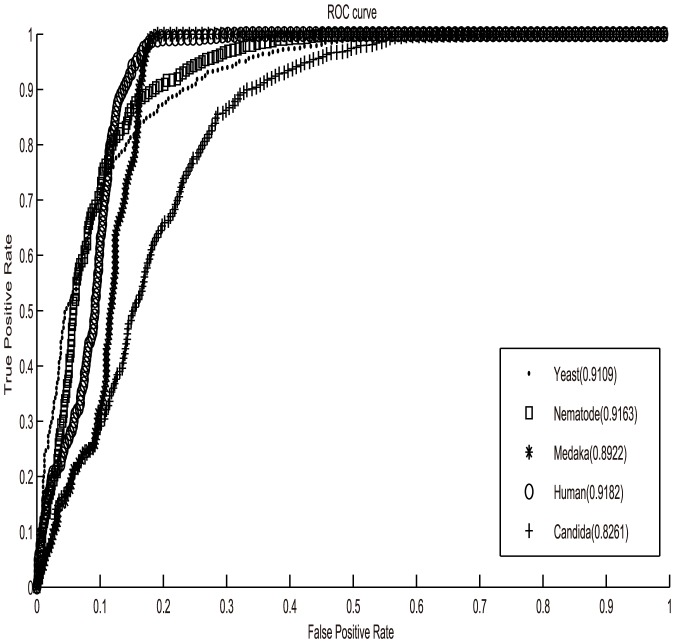
Classification performance of the SVM based on nucleosome positioning information model for five organisms. Values in parentheses indicate the area under the receiver operating characteristic curve (AUC) for each organism.

To evaluate the performance of our model, the averaged ROC curves of our new model were shown in Figure 1 with a mean AUC value equal to 0.8927. The result showed that the prediction accuracy of our model was significantly higher than the previous methods above (Table 3). Besides, it was shown that except for Candida and Medaka, the AUC values of other three species were all above 0.9, which meant the abilities of our model for these three species (Human, Nematode, Yeast) were excellent. However, even for Candida and Medaka, the AUC values have been improved from 0.766 to 0.8261, 0.884 to 0.8922 respectively. The result again suggests that the proposed two index vectors can efficiently capture some aspects of the sequence-dependent affinity of the histone octamer. Meanwhile, the geometrically transformed Tsallis entropy is a valid indicator to extract the nucleosome positioning information.

**Table 3:** AUC values of our model compared with previous work.

	Human	Medaka	Nematode	Candida	Yeast	Average
Segal(ver.3)	0.694	0.516	0.708	0.722	0.764	0.681
Segal(ver.2)	0.684	0.53	0.717	0.752	0.804	0.697
Segal(ver.1)	0.487	0.565	0.492	0.51	0.514	0.514
Miele	0.333	0.508	0.319	0.425	0.313	0.379
Gupta(Linear)	0.611	0.605	0.696	0.678	0.802	0.678
Gupta(Quadratic)	0.611	0.605	0.697	0.682	0.794	0.678
Gupta(Cubic)	0.596	0.634	0.702	0.673	0.799	0.681
Gupta(RBF1)	0.695	0.705	0.743	0.69	0.811	0.729
Gupta(RBF5)	0.641	0.659	0.744	0.703	0.796	0.709
Gupta(RBF10)	0.657	0.642	0.736	0.705	0.798	0.707
Zhang et al. [17]	0.872	0.884	0.836	0.766	0.831	0.838
Our model	0.9182	0.8922	0.9163	0.8261	0.9109	0.8927

### Genome-wide prediction of nucleosome in S.cerevisiae

The average nucleosome occupancy profile along the S.cerevisiae genome can be obtained based on nucleosome occupancy model. To illustrate the validity of our approach, the comparisons with some experimental results should be done. In 2008, Kaplan et al. [9] compared the nucleosome occupancy of extracted 20000 bp typical genomic regions of S.cerevisiae under different growth conditions (YPD, ethanol and galactose) in vivo with vitro. The average nucleosome occupancy profile of the same extracted region is also done in this work.

**Figure 2:**
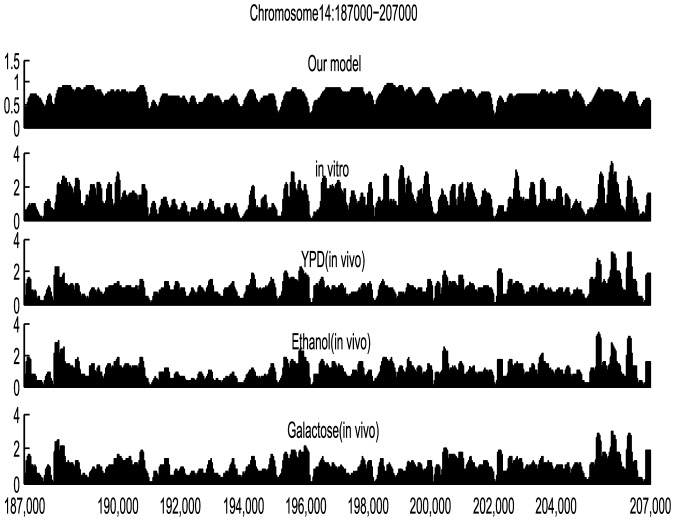
The average nucleosome occupancy predicted by our model compared with some experimental results for a typical 20,000-bp-long genomic region. The top line represents the average nucleosome occupancy predictions from our model. The second graph represents the experimental map in vitro. The third, fourth and fifth graphs represent in vivo experimental maps for three growth conditions (YPD, galactose and ethanol), respectively.

Examining Figure 2, we found a high similarity between the average nucleosome occupancy profile predicted by our model and experimental map of nucleosome occupancy in vitro (0.7116), in vivo growing in ethanol (0.5654). According to peak detection model, peaks along average nucleosome occupancy profile are critical positioning signals. Comparing these five graphs, peaks match well, which provides us basis for the accurate nucleosome positioning. These results imply that our model has an excellent predictive ability on recognizing the nucleosome-enriched and nucleosome-depleted regions in the S.cerevisiae genome.

We have summarized some existing nucleosome maps of Saccharomyces cerevisiae [5], [6] and compared our result with their publications. In Segal's work [5], they provided the probability that any basepair is covered by nucleosome and nucleosome positions with higher probability (>0.2). All data can be downloaded from their website (http://genie.weizmann.ac.il/pubs/nucleosomes06). In the work of Yuan et al. [6], the researchers constructed a N-score model
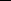
a wavelet analysis based model for predicting nucleosome positions from DNA sequence information.

**Figure 3:**
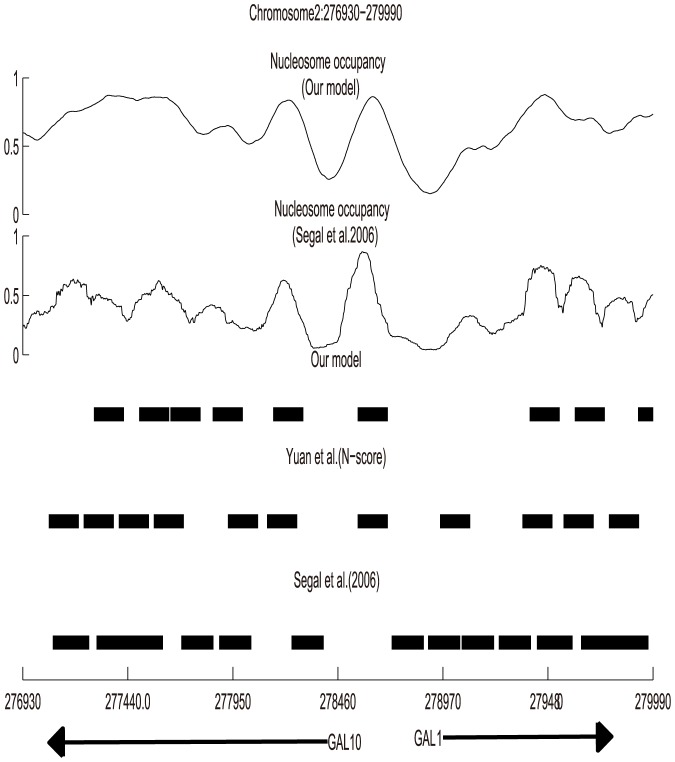
Detailed view of the predictions of intrinsic nucleosome organization along GAL1-10 locus (Chromosome II: 276930-279990) and comparison to Segal's and Yuan's results. The first top two line are nucleosome occupancy profile predicted by our model and Segal's. The black boxes in the third, fourth and fifth line are the identified nucleosome positions in this study, Yuan (N-score) [6] and Segal et al. [5], respectively.

Figure 3 shows two different average nucleosome occupancy profile of the GAL1-10 locus (chromosome 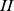: 276930-279990) in the first top two panels: the Segal's average binding score and our average nucleosome occupancy. Nucleosome predictions by our model, Yuan et al. [6], Segal et al. [5] were listed in the third, fourth and fifth panel respectively. The figure shows that the average nucleosome occupancy profile of our model and Segal's are apparently similar with a correlation of 0.7846. Besides, comparing three nucleosome positioning maps, we found a significant correspondence. High degree of similarity was seen between our predictions and Yuan's result. Eight nucleosomes of Yuan's eleven predictions were identified with only a small shift. When compared with Segal's map, only seven nucleosomes among Segal's predictions were identified.

Here, we also downloaded the predicted nucleosome positions with Yuan (N-score) [6] and the 2003 version of yeast genome (http://bcb.dfci.harvard.edu/). We presented a complete nucleosome positions map along Chromosome 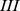 and compared with Yuan's result. A set of 1281 central locations of well-positioned nucleosomes along Chromosome 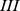 were listed in Yuan's result while in our work, a set of 1053 nucleosome positions have been predicted. In order to evaluate our predictions more intuitively, we defined two parameters. One is the fraction of the positions in the Yuan's work that are within X nucleotides of a predicted position. Another is the fraction of the positions in our work that are within X nucleotides of a predicted position by Yuan et al.

The result shows that nearly all the central positions of nucleosomes 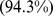 in Yuan's predictions are within 147 bp (the length of one nucleosome) of our results, in other words, 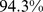 of Yuan's result were overlapping with our predictions. In addition, 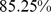 of our predicted nucleosomes are within 147 bp of Yuan's result, which means the majority of our predictions are valid. Both these two fractions significantly exceeded random prediction. These results indicate that, taking the work of Yuan et al. [6] as reference, our model is valid in the predictions of nucleosome positions along genome.

### Model comparisons

In recent years, with the advances in high-throughput DNA sequencing technology, a number of high-resolution genome-wide maps of nucleosomes in S.cerevisiae have been derived experimentally. However, nucleosome positions are determined by numerous factors, among which the DNA sequence has been proved to play an important role. Thus, some prediction algorithms based on DNA sequences are also proposed. Therefore, it requires an objective and impartial comparison of different nucleosome maps. Here, we presented six high-resolution genome-wide maps of S.cerevisiae nucleosome positions (five published [5]–[7], [11], [12], and one published here (Table 4, Table 5). The experimentally measured maps from multiple labs and detection platforms and the nucleosome positions achieved by mathematical and physical algorithms are all listed.

**Table 4:** Summary of experimental methods.

Model names	Strains/Culture	Platform	Detection strategy	Number/Resolution
Albert(H2A.Z) [12]	BY4741/rich media	Pyrosequencing	Chip-Seq	∼10,000/∼4 bp
			Length: ∼25 bp	
Mavrich(H3/H4) [11]	BY4741/YPD	The Roche GS20	Chip-Seq	54,753/∼1 bp
		454 Life Sciences	Length >100 bp	
Lee(HMM) [7]	BY4741/YPD	Affymetrix	HMM	70,871/4 bp

**Table 5:** Summary of algorithms.

Model names	Training dataset	Extraction strategy	Detection strategy	Number(ChrIII)
Our model	Yuan et al. [4]	Tsallis Entropy	Peak Detection	1053
Yuan(N-score) [6]	199 nucleosomes [5]	N-score	Threshold method	1281
	296 linkers [4]			
Segal(0.2) [5]	199 nucleosomes	Apparent Free Energy	HMM	2068
Segal(0.5) [5]				403

**Figure 4:**
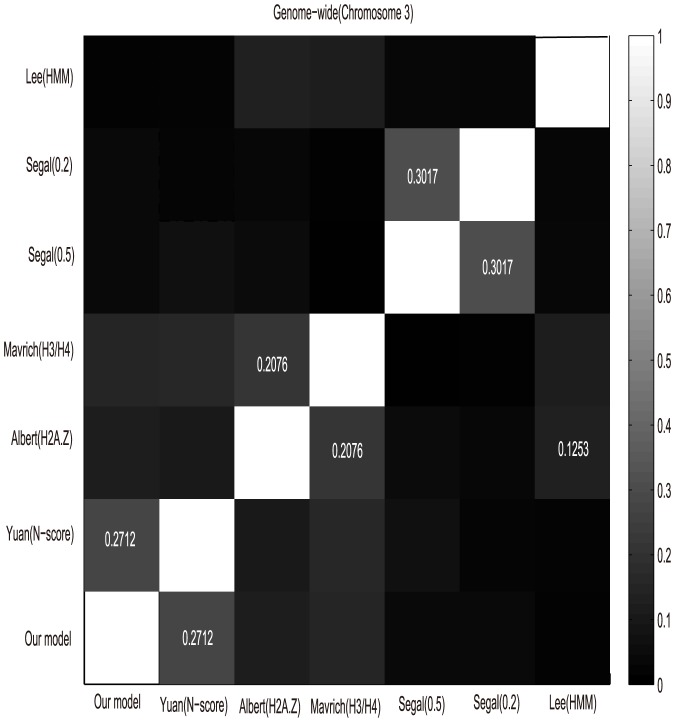
The correlation coefficient between cross-platform nucleosome positioning along Chromosome III. In the heat maps, the marked number represents corresponding correlation coefficients between datasets.

We compared the three experimentally determined nucleosome maps [7], [11], [12] with those obtained by score-dependent procedures [5], [6]. In order to resolve the disagreement between datasets, packaging DNA is represented by 1 and DNA without nucleosome to 0. As a result, a set of binary data corresponding to the yeast's Chromosome 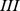 are constructed. To get a rough assessment of the discrepancies and consistency between the six datasets, we calculated the Pearson correlation coefficients between the nucleosome positioning maps along Chromosome 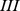, see Figure 4. Even if the purpose of these six experiments are all to get genome-wide nucleosome map, their focus, priorities and platforms are different. And there is no standard nucleosome positioning map now. As a result, all six maps showed only a modest correlation with maximal correlation coefficient of 0.2712(Our model and Yuan(N-score)). Here, we don't take the correlation between Segal(0.2) and Segal(0.5) into consideration because they are two results in one paper [5]. Notably, we found that the six maps can be divided into four groups: Our model 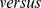 Yuan(N-score), Albert(H2A.Z) 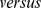 Maverich(H3/H4), Segal(0.5)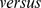 Segal(0.2), Lee(HMM).

Firstly, three listed algorithms [5], [6] depend only on the DNA sequence, while the nucleosome positions in vivo are determined by the combination of many factors. Thus, it is not necessary to mind the difference between these three predictions and experimentally determined maps. However, our result show that nucleosome positions in vivo depend, at least partially, on DNA sequences. Comparing nucleosome maps determined experimentally and predicted maps, the modest correspondence can be attributed, in part, to additional factors that influence nucleosome positioning. Besides, the three prediction algorithms are only trained on a small number of nucleosomal and Linker DNA sequences (Table 4, Table 5), which allowed only a rough estimation of the parameters in their algorithms, such that the model scores are also only of approximate nature.

However, Albert(H2A.Z)(Mavrich(H3/H4)) were constructed by direct sequencing of the nucleosomal-sized DNA fragments with H2A.Z (H3 or H4) containing nucleosomes. In the work of Lee et al. [7], the chromatin was digested to mononucleosomal DNAs by MNase. Then, as a control, the corresponding nucleosomal DNA fragments and fragmented genomic DNA were hybridized to tiling microarrays with four base pair resolution. For nucleosome positions detection, Lee(HMM) used HMM to obtain the nucleosome positions. Thus, the different experiment procedure should influence not only on the analyzed data but also on the raw data.

From Table 5, we learned that both Yuan(N-score) and Segal(0.2), Segal(0.5) used the same positive training dataset, but Yuan(N-score) also constructed a negative training dataset consisting of 296 Linker DNAs. However, we can find that Yuan(N-score), Segal(0.2) and Segal(0.5) are all trained on the experimentally extracted nucleosomal and linker DNA sequences. In our study, the training dataset is from Yuan et al. [4]. In Yuan's work, they designed a microarray to score 13742 50-bp fragments from chromosome 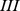. We ranked these sequences according to scores and chose 1000 fragments with the highest scores as the positive dataset, 1000 fragments with the lowest scores as the negative dataset.

Besides, they also differed in the methods of extracting nucleosome positioning information and detecting nucleosomes. For the extraction of nucleosome positioning information, Yuan(N-score) involves wavelet decomposition of the three point average dinucleotide frequencies using the Haar wavelet. Segal's work defined a function called the apparent free energy to compute the probability that a sequence S is generated by considering the space barrier and the competition of neighboring nucleosomes. While in our study, we proposed a new nucleosome positioning information model by proposing the geometrically transformed Tsallis entropy to extract the conservation of A/T-riched and G/C-riched dimeric and trimeric motifs along arbitrary DNA duplex. These three algorithms extracted nucleosome positioning information from different aspects.

**Figure 5:**
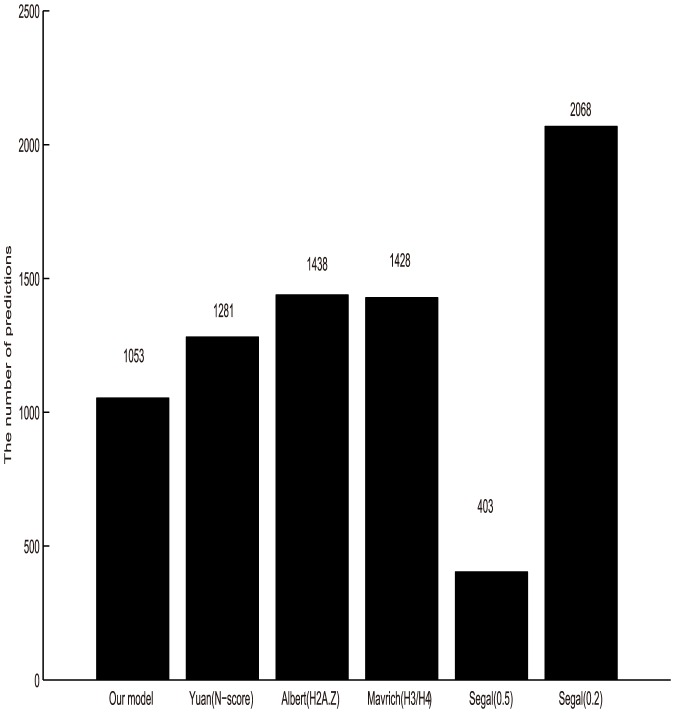
The number of predicted nucleosomes across six maps (This study, Yuan(N-score), Albert(H2A.Z), Mavrich(H3/H4), Segal(0.5), Segal(0.2)) along Chromosome III.

**Figure 6:**
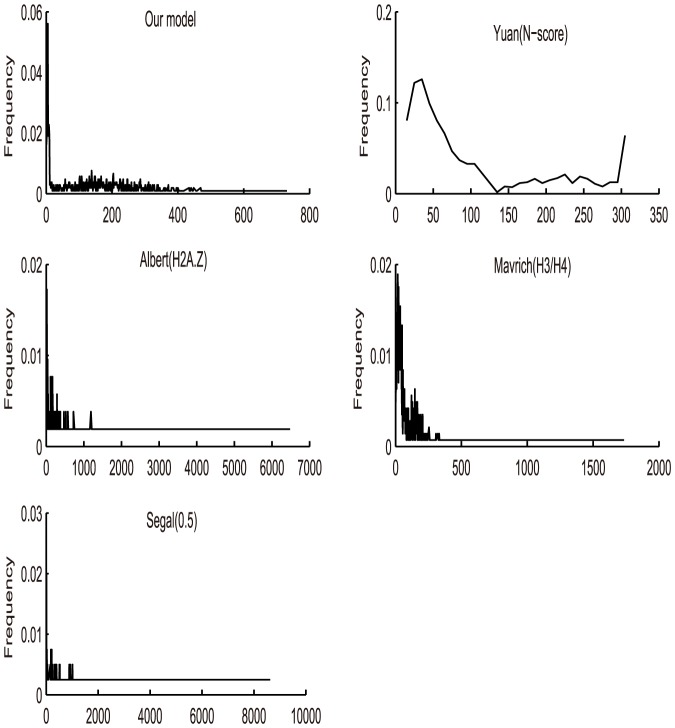
Frequency of linker lengths across five maps (This study, Yuan(N-score), Albert(H2A.z), Mavrich(H3/H4), Segal(0.5)).

Apart from the different nucleosome positioning information model and training set, we also compared the nucleosome detection methods. In Segal's work, they constructed a nucleosome-DNA interaction model and used the popular hidden Markov model (HMM) to obtain the final nucleosome positions. While, Yuan(N-score) proposed a model called N-score to measure the probability of arbitrary sequence to be nucleosome. They also used a stepwise procedure to select predictors and estimate the corresponding coefficients using a program in SAS for the distinguish of nucleosomes. In this study, based on the concept of relative distance, we obtained the probability of any DNA sequence occupied by nucleosomes. At last, we presented a peak detection model with two-step filtration to get the final genome-wide map. The advantage of our peak detection model is that it assigns nucleosome positions in a score-dependent fashion, i.e. our maps are dependent only on local score maxima, while the procedure of Segal et al. and Yuan et al. require the determination of additional parameters, such as the coefficients in both Yuan's stepwise procedure and Segal's HMM to ensure comparability. Besides, it has been suspected that when HMM is trained on the nucleosomes with a uniform distribution, it may cause the continuity of such uniform, even in the nucleosome-free regions (NFR). As a result, HMM will lead to the over-estimation of the uniformity and density of nucleosomes along genome-wide sequence [13]. We validated this idea by the comparison of these six maps, see Figure 5 and Figure 6.

This paper presents a new sequence-based nucleosome positioning method. Furthermore, we will show the validity of our new model by comparing the performance of our model with that of the Lee(HMM), Segal(0.2), Segal(0.5) and Yuan(N-score). We downloaded the nucleosome map of 99 nucleosome positions determined at 11 individual loci as the reference map1. Besides, we also compiled a new genomic nucleosome positions from Albert(H2A.z) and Mavrich(H3/H4) by logAND as reference map2.

**Figure 7:**
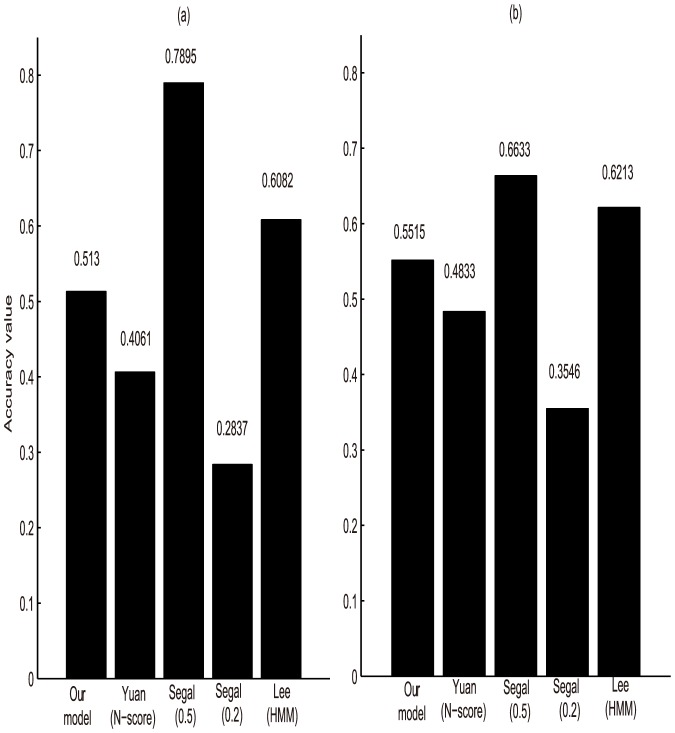
Model-specific values of accuracy. The accuracy values is plotted for each model. The bars indicate the measured accuracy value. (a) Accuracy values using the map1 as the reference. (b) Accuracy values using the map2 as the reference.

Four parameters have been proposed to measure the model's performance: total accuracy (Accuracy), the sensitivity (Sensitivity), positive predictive value (Precision) and Matthews correlation coefficient (MCC). Here, this paper redefines Accuracy to measure the performance of different models in nucleosome positioning along the genomic sequence. TP represents the number of correctly predicted positions covered by nucleosome in the reference map. TF is the number of correctly predicted positions uncovered by nucleosome. Similarly, FP and FN represent the number of incorrectly predicted positions covered or uncovered by nucleosome in the reference map respectively. Here, an Accuracy value of 1 indicates perfect prediction, i.e. all predicted nucleosomes are predicted with zero positional error comparing with the reference map. The results are summarized in Figure 7.

In fact, all five models show only a modest predictive power with maximal Accuracy values of 0.7895 (Figure 7a) and 0.6633 (Figure 7b). Besides, we found that the correspondence between the experimental maps are limited(Lee(HMM) versus Reference map1: 0.6082, Lee(HMM) versus Reference map2: 0.6213). The Accuracy values across five maps are all changed when the reference map changes. This can be interpreted that the experimentally mapped nucleosome positions exhibit different due to the different focus, emphasis and platforms. Thus, in this study, we can not find a standard nucleosome map as the training dataset, but to choose the dataset which performs best after many trials. This may contribute to the low Accuracy value of our model. However, our new model outperforms the existing models (Yuan(N-score), Segal(0.2)). Comparing the two results of Segal's model, we can find that the result of Segal(0.5) was significantly higher than Segal(0.2) in two experiments. Perhaps, if the researchers want to locate nucleosomes by HMM, they need to filter predictions more strictly, so as to improve the accuracy. In summary, our results confirm the idea that the DNA sequence determines nucleosome positions in vivo in concert with other factors. Moreover, our model has a good performance to capture some aspects of the sequence-dependent affinity of the histone octamer.

## Discussion

Nucleosome positioning is an important chromatin feature that regulates gene expression. However, the precise mechanism has not been fully understood. Many researches have revealed that nucleosome positioning is not determined by any single factor but rather by the combined effects of multiple factors including DNA sequence, DNA-binding proteins, nucleosome remodelers and the RNA polymerase II transcription machinery. By constructing a probabilistic model to represent the DNA preferences of nucleosomes, Segal et al. [5] demonstrated that nucleosome organization is encoded in eukaryotic genomes and explained 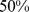 of the in vivo nucleosome organization. Here, we provided another perspective to study the role that DNA sequence preferences play in nucleosome organization.

Firstly, we calculated the correlation coefficients between four nucleotides and the nucleosome occupancy across five organisms. The result clearly showed that four nucleotides can be divided into two categories donated by W and S. Secondly, inspired by the pioneering work of Trifonov [15], which was the AT-riched and GC-riched dimeric and trimeric motifs were contributed to nucleosome organization, we would like to further explore the role that A/T-riched and G/C-riched dimeric and trimeric motifs plays in nucleosome positioning by defining two index-vectors. The first index-vector extracted the frequencies of A/T-riched and G/C-riched dimeric motifs (WW and SS) and achieved high correlations with nucleosome occupancy across five organisms.

**Figure 8:**
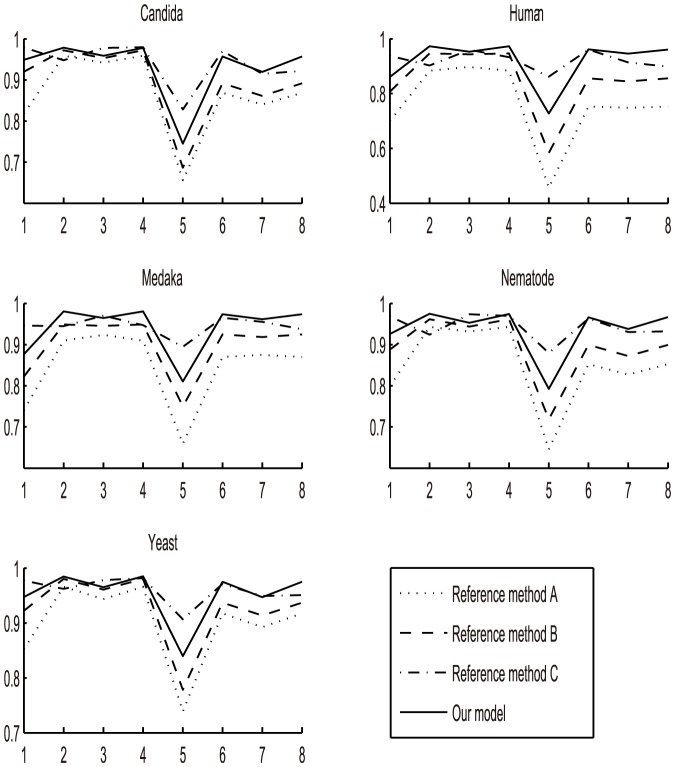
Comparisons of four methods (Our method, Reference method A, Reference method B, Reference method C) across five organisms. The point represents corresponding correlation coefficients between each vector component and nucleosome occupancy.

Next, we sought to explain why the second index-vector (i.e. 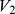) is selected to describe the distribution of A/T-riched and G/C-riched trimeric motifs. Here, we listed another three common reference methods to illustrate the superiority of the proposed method: (A) The first method is put forward from the opposite direction of our method, which is the probability vector with the ratio of total occurrences of the A/T-riched and G/C-riched trimeric motifs to that of the nucleotides occur once or never appear (i.e. 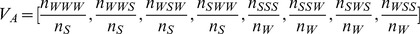). (B) The frequencies of A/T-riched and G/C-riched trimeric motifs (i.e. 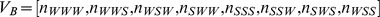). This method of extracting sequence information is very common in many studies. Peckham et al. [8] firstly transformed each DNA sequence into a 2,772-element vector, in which each entry is a normalized count of the occurrences of a particular k-mer or its reverse complement, for k = 1 up to 6 to train SVM for the discrimination of nucleosomal and linker DNAs of Saccharomyces cerevisiae. Afterwards, Gupta et al. [21] applied the same way on the dataset of Human. Both two methods have achieved appreciable results. (C) This method is similar to the dinucleotide absolute frequency proposed in the study of Zhang et al. [17]. It is defined as the ratio of total occurrences of the trinucleotide to that of the first dinucleotide composing it (i.e. 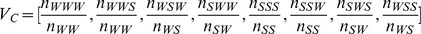). We then performed a method selection step to compare these four methods in order to identify which method is most suitable for our study by calculating the correlation coefficients between the four transformed vectors and nucleosome occupancy across five organisms (Figure 8).

We note that the five comparison charts showed the same result. Obviously, our method achieved higher correlations with nucleosome occupancy than the reference method A and B for all eight components. When compared with the reference method C, some dimensions in 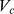 showed better performance than our model, such as 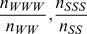, while some components were significantly less correlated with nucleosome occupancy, even worse than the preference method A and B. Thus our proposed index-vector showed its high and stable level of correlations with nucleosome occupancy across five organisms and so was selected as the description of the distribution of A/T-riched and G/C-riched trimeric motifs. In general, the result indicated that both two proposed index-vectors had strong correlations with nucleosome occupancy, at least 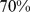 of nucleosomal DNAs can be explained by the two index-vectors. Across five organisms, some common conclusions can be obtained. The fifth vector component (i.e.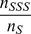) showed the smallest correlation with nucleosome occupancy for all five organisms, while the first vector component (i.e.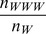) was also less correlated. It may be interpreted that the trinucleotides which are the combination of two A/T or two G/C steps are more important for promoting nucleosome positioning among all trinucleotides. The repetitive occurrence of CAG/CTG is known to form a stable nucleosome DNA. In this way, we eliminated the nucleotide differences among five organisms and proposed two uniform index-vectors to present the distribution of A/T-riched and G/C-riched dimeric and trimeric motifs.

To gain more direct evidence for the importance of our index-vector to intrinsic nucleosome occupancy, we calculated Pearson correlation coefficient between the proposed index-vector and nucleosome occupancy along genomic sequence of Saccharomyces cerevisiae. Here, we used the in vitro data provided by Kaplan et al. [9] and selected 107630 bp region along chromosome 14. The typical 20,000-bp-long genomic region in Figure 2 is included in this region. A 147-bp sliding window was used to scan chromosome 14 in 1-bp step. In order to get the index-vector for each position along the selected region, we adopted the following measures. For the index-vector of position i, we counted index-vectors of sequences starting at position i-146 to i, which will cover position i if the sequence is nucleosomal DNA. And the average index-vector was taken as the index-vector for position i. Then, the correlation coefficient of index-vector and nucleosome occupancy was calculated. The result shows that the first vector component 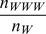 correlates highly with nucleosome occupancy in vitro (R = 0.7048) and the second and fourth vector components are less correlated. In the work of Desiree et al. [28], both G+C and AAAA were identified as two features correlating most highly with nucleosome occupancy in vitro (R = 0.71 and 0.63 respectively) among the selected 14 features. We note that the first vector component 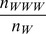 shows the near level of correlation with nucleosome occupancy in vitro. It suggests that 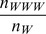 itself is a good predictor for nucleosome occupancy. And, 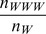 may be an important influencing factor of nucleosome organizations for Saccharomyces cerevisiae.

To explore the relationship between our extracted index-vector and structural features, we examined index-vector and structural features in an independent data set in Kaplan's work [9], in which nucleosomes were assembled with synthetic 150-mer sequences (both microarray and sequencing datasets). Here, we presented twelve structural features: slide, rise, clash strength, free energy, tip, enthalpy, roll, tilt, twist, wedge, propeller twist and entropy change, which characterize various structural aspects of DNA sequences. The structural values were calculated as the average over each provided sequence:
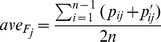
(18)Where 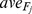 is the average value of the property 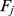, 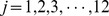 and 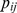 and 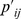 are the corresponding structural values of the dinucleotide at position 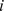 along DNA strand and its reverse complement strand for the property 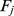.

Even the synthetic 150-mer nucleosome occupancy data was described by Kaplan et al. [9] as noisier than the yeast genomic DNA occupancy data, both the synthetic oligonucleotides measured by microarray and synthetic oligonucleotides measured by sequencing have been confirmed displaying the same global trends with yeast genomic DNA, both in vitro and in vivo from the angle of DNA structural parameters [28]. Next we would like to explore to what extent the index-vector dictate nucleosome structure and the Pearson correlation coefficients between the 12 structural properties of DNA sequences and index-vector were calculated.

**Figure 9:**
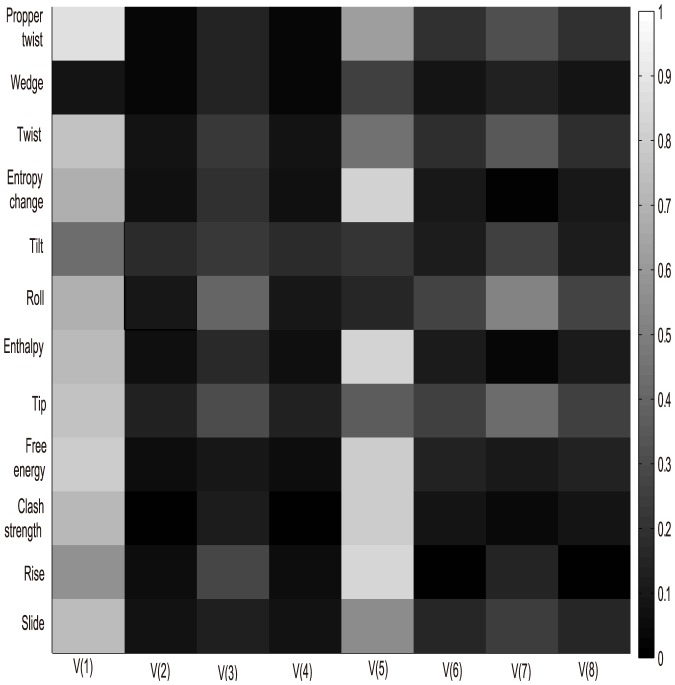
Graphic illustration of the correlation of each of the twelve structural features with index-vector (Sequence data is the synthetic 150-mer nucleosome occupancy data measured by microarray from Kaplan et al. [9]).

**Figure 10:**
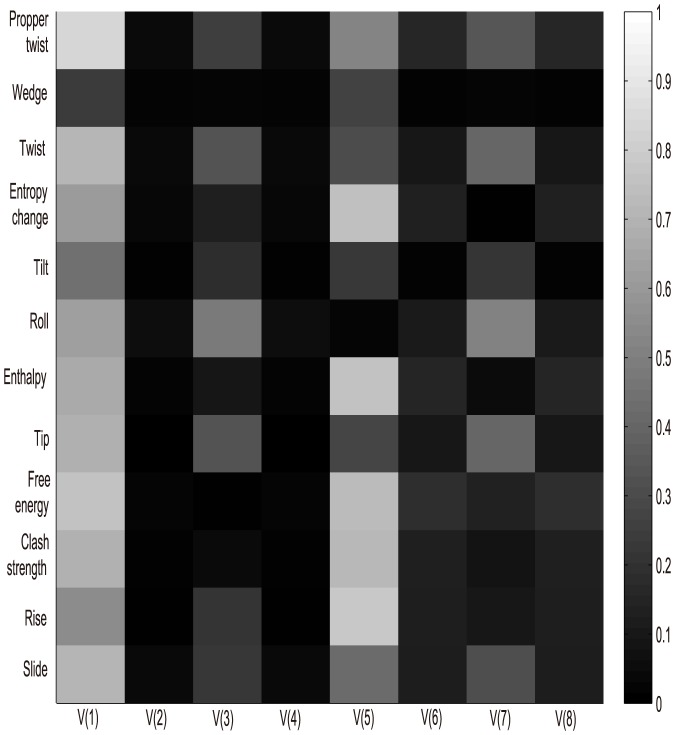
Graphic illustration of the correlation of each of the twelve structural features with index-vector (Sequence data is the synthetic 150-mer nucleosome occupancy data measured by sequencing from Kaplan et al. [9]).

In Figure 9 and Figure 10, V is the second index-vector in function (4) and V(i) denotes the 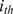 dimension of vector V. Both two figures showed that the proposed index-vector is not fully independent with selected structural features. Both the first and fifth vector components showed highly correlated with all the 12 structural features in the two datasets. While the second and fourth vector components have the worst correlation with the structural features. This can be explained that the distribution of trinucleotides made up of three A/T or C/G are more important than trinucleotides, which are the combination of two A/T or C/G steps in the influence of nucleosome structure. Gan et al. have shown that structural properties of DNA sequence would directly determine nucleosome occupancy [14]. Meanwhile, this also illustrates the importance of our index-vector to nucleosome positioning from the structure-based perspective. Here, we pointed out the first vector component 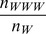, which showed its high correlations with both nucleosome occupancy along genomic sequence and twelve structural features.

The importance of 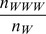 may also be explained from the following several aspects. Firstly, 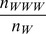 depicts the distribution of WWW (W is A or T) and the appearance of WWW will limit the frequency of C+G, which has shown its high correlation with nucleosome occupancy in the work of Desiree et al. [28]. Besides, from the above analysis, we can find that this single parameter affects nearly all aspects of DNA structure, which provides evidence of another angle for its importance on nucleosome organization. Moreover, ploy(dA:dT) tracts have been proved being important signal for nucleosome packaging and the occurrences of WWW tend to increases the frequency of poly(dA:dT)-like tracts.

Then, the geometrically transformed Tsallis entropy was introduced to describe the total ordering of DNA sequences from the point of depicting the distribution of A/T-riched and G/C-riched dimeric and trimeric motifs along DNA sequence. When calculating the geometrically transformed Tsallis entropy of nucleosomal and linker DNAs across five organisms, the average values of the eight entropies of nucleosomal DNAs were all obviously lower than linker DNAs for five organisms. This suggests A/T-riched and G/C-riched dimeric and trimeric motifs are better ordered along nucleosomal DNAs than linker DNAs, which may be related with the 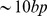 periodicity of WW (W = A or T) and SS (S = C or G) in nucleosome DNA regions. What's more, the validity of our model can also be verified from the performance of distinguishing known nucleosomal and linker DNAs compared with the results of Segal et al. [5], [9], [22], Miele et al. [20], Gupta et al. [21] and Zhang et al. [17].

Moreover, our study offered an idea to describe average nucleosome occupancy at each basepair along genomic sequences from the point of relative distance. The effectiveness of this method has been proved from the following two aspects. Firstly, when tested on a randomly extracted dataset consisting of nucleosomal DNAs with fixed-length and linker DNAs with different lengths. The result indicates the effectiveness of this method is not affected by the different lengths of linker DNAs. Secondly, the genome-wide profiles of average nucleosome occupancy is highly correlated with both Kaplan's experimental map and Segal's result. The peaks of average nucleosome occupancy profile well correspond to nucleosome regions and the valleys match nucleosome-depleted ones. From the above, the relative distance is a valid index describing nucleosome occupancy and the average nucleosome occupancy profile can directly represent the nucleosome distribution along genomic sequences.

Besides, a peak detection model was introduced to locate the accurate nucleosome positions with the consideration of competition for space between two neighboring nucleosomes. Furthermore, we defined two fractions to evaluate the accuracy of our predicted nucleosome positions. The result shows that 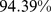 in Yuan's result were overlapping with our predictions. Our method shows the important role that DNA preference plays in nucleosome positioning and further widen the idea of nucleosome positioning research.

## Conclusion

We have established a simple and efficient nucleosome positioning model consisting of nucleosome positioning information model, nucleosome occupancy model and peak detection model by describing the regularity of A/T-riched and G/C-riched dimeric and trimeric motifs along sequence. The values of AUC across five organisms (Human, Medaka, Nematode, Candida and Yeast) significantly outperformed the previous works (Table 3). The index-vector component 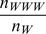 may be an important factor for nucleosome positioning of Saccharomyces cerevisiae, which depicts the distribution of WWW (W is A or T). The analysis shows that it highly correlates with nucleosome occupancy and some structural properties. Maybe, its importance on nucleosome organization can also be interpreted by the fact that it increases the frequency of poly(dA:dT)- tracts. Besides, with the nucleosome occupancy model and peak detection model, we also gave the average nucleosome occupancy profile as well as the precise locations of nucleosome along S.cerevisiae genome. By comparing with some published results [5], [6], [9], the conclusion can be drawn that our method is valid in predicting nucleosome occupancy and positions along genomic sequence. Our findings suggest that the distribution of A/T-riched and G/C-riched dimeric and trimeric motifs along sequence have a significant influence on chromatin structure.
